# Widely Targeted Lipidomics and Transcriptomics Analysis Revealed Changes of Lipid Metabolism in Spleen Dendritic Cells in Shrimp Allergy

**DOI:** 10.3390/foods11131882

**Published:** 2022-06-25

**Authors:** Shanfeng Sun, Jiangzuo Luo, Hang Du, Guirong Liu, Manman Liu, Junjuan Wang, Shiwen Han, Huilian Che

**Affiliations:** Key Laboratory of Precision Nutrition and Food Quality, Key Laboratory of Functional Dairy, Ministry of Education, The 2115 Talent Development Program of China Agricultural University, College of Food Science and Nutritional Engineering, China Agricultural University, Beijing 100083, China; 18302432883@163.com (S.S.); luo534268@163.com (J.L.); d958380069@163.com (H.D.); liuguirong1998@cau.edu.cn (G.L.); 15810888073@163.com (M.L.); wangjunjuan93@163.com (J.W.); shiwen0414@163.com (S.H.)

**Keywords:** shrimp allergy, lipid metabolism, dendritic cells, immune metabolism, lipidomics, transcriptomics

## Abstract

Shrimp allergy (SA) is pathological type 2 inflammatory immune responses against harmless shrimp protein allergen, which is caused by complex interactions between dendritic cells (DCs) and other immune cells. Lipid metabolism in different DCs states are significantly changed. However, the lipid metabolism of spleen DCs in SA remain ambiguous. In this study, we established a BALB/c mouse shrimp protein extract-induced allergy model to determine the lipid profile of spleen DCs in SA, and the molecular mechanism between lipid metabolism and immune inflammation was preliminarily studied. Spleen DCs were sorted by fluorescence-activated cell sorting, and then widely targeted lipidomics and transcriptomics analysis were performed. Principal component analysis presented the lipidome alterations in SA. The transcriptomic data showed that *Prkcg* was involved in lipid metabolism, immune system, and inflammatory signaling pathway. In the correlation analysis, the results suggested that *Prkcg* was positively correlated with triacylglycerol (Pearson correlation coefficient = 0.917, *p* = 0.01). The lipidomics and transcriptomics integrated pathway analysis indicated the activated metabolic conversion from triacylglycerol to 1,2-diacyl-sn-glycerol and the transmission of lipid metabolism to immune inflammation (from triacylglycerol and ceramide to *Prkcg*) in SA spleen DCs, and cellular experiments in vitro showed that glyceryl trioleate and C16 ceramide treatment induced immune function alteration in DCs.

## 1. Introduction

Allergic diseases are an increasing health and economic problem all over the world. Food allergy impacts 1–2% of adults and 5–8% of infants and young children worldwide [1]. Shellfish belongs to the eight major food allergens and is a frequent elicitor of food-induced anaphylaxis in Asia and central Europe [2,3]. It is estimated that 2% of Americans have shellfish allergies [4]. Shellfish is the most common cause of allergic reactions, and shellfish allergy accounts for the majority of emergency department visits related to severe food allergies, making shellfish allergy an urgent public health problem [5,6]. Presently, the methods for determining allergens in food mainly include methods based on protein and methods based on nucleic acid. Methods based on protein can be divided into immunoanalytical methods (such as enzyme-linked immunosorbent assay (ELISA), Western blot, lateral flow immunoassay, biosensor, and protein chips) and mass spectrometry. Methods based on nucleic acid mainly include polymerase chain reaction (PCR) and real- time PCR (qPCR), loop-mediated isothermal amplification, and DNA microarray [7].

The main effector cells involved in initiating and establishing allergic responses are antigen-presenting cells (DCs), T helper 2 (Th2) cells, IgE-producing plasma cells, and mast cells. When allergen enters the body through epithelial cells for the first time, it can be modified by DCs and presented to naive CD4^+^T cells, which promotes the differentiation of naive CD4^+^T cells into Th2 cells, secretes a large number of Th2 cytokines such as interleukin −4 (IL-4), and induces B cells to produce allergen-specific IgE (sIgE), which binds to immunoglobulin receptor I (FcεRI) with high affinity on mast cell membranes. When there is re-exposure to the same allergen, the allergen directly cross-links with the IgE-FcεRI complex bound on the membrane of mast cells, activates degranulation of mast cells, and releases histamine and inflammatory mediators [8]. It can be concluded that DCs are upstream of the initiation and maintenance of food allergy and are a bridge between antigen and naive CD4^+^T cells in the pathogenesis of food allergy. DCs can regulate the differentiation of CD4^+^T into Th1 or Th2 and exert immune function through the expression of surface molecules, such as co-stimulating molecules CD40, B220, and CD68, thus inducing immune skews [9]. Therefore, it is of great significance to study the mechanism of DCs’ immune function in food allergy, and to develop new prevention and treatment methods of food allergy based on DCs.

Significant differences in lipid metabolism can be found in different DC states (resting or activated, immunogenic or immune tolerance) in different physiological and pathological conditions (such as allergy [10], tumor [11], obesity [12], and asthma [13]). Reprogramming of lipid metabolism plays a vital role in establishing and maintaining the phenotype and function of DCs [14], and the pathological conditions can be regulated by regulating lipid metabolism pathways. Yao et al. [15] reported that oleoylethanolamide downregulates TLR4/NF-κB, the classical pathway leading to DCs’ maturation, through the activation of TRPV1 and AMPK. Activated AMPK increases fatty acid oxidation (FAO) by inhibiting CPT I, and melanoma induces FAO in DCs via the Wnt5a-β-catenin PPAR-γ signaling pathway and upregulates the expression of CPT 1A in the tumor microenvironment [16]. Moreover, the induction of Th2-dependent eosinophilic airway inflammation can be prevented by activation of PPAR-γ [17]. However, the lipid metabolism of spleen DCs in SA remains ambiguous.

This study investigated the lipid profile of spleen DCs in SA by widely targeted lipidomics and transcriptomics. Furthermore, we analyzed the correlation between lipid metabolism and immune inflammation of DCs in SA, and the molecular mechanism between lipid metabolism and immune function was preliminarily studied. These data may provide evidence for further molecular mechanism study of the immune metabolism of DCs in SA and enable new treatment targets/strategies. Since DCs are upstream of food allergy, the regulation of DC immune function has great therapeutic potential for food allergy. The results of the present study will make an important contribution to mitigating SA from upstream of SA by regulating the DC immune function through lipid metabolism.

## 2. Materials and Methods

### 2.1. Experimental Animals

Forty-eight four-week-old female specific pathogen-free BALB/c mice (14–18 g) were purchased from Vital River Laboratories (Beijing, China, SYXK (Jing) 2020-0052) and housed at 23 ± 3 °C and 40–70% relative humidity with 12 h light/dark cycle. Mice had free access to distilled water and a shrimp protein-free diet. Mice were adaptively fed for seven days before the experiment. China Agricultural University’s Experimental Animal Ethics Committee reviewed and approved the animal study (No. AW70101202-4-1).

### 2.2. Experimental Scheme

Mice were randomly divided into control group (CK) and SA group (24 mice in each group). On day 0, 7, 14, 21, and 28, the mice received 250 μL saline or 250 μL of shrimp protein extract (4.8 mg/mL) including 10 μg of CT by gavage in CK or SA, respectively [18,19]; 250 μL of shrimp protein extract (24 mg/mL) was used for challenge by gavage on day 42.

### 2.3. sIgE and sIgG1 Analysis

On day 35 of the experiment, blood samples were collected from the orbital venous plexus. After standing overnight at 4 °C, the samples were centrifuged at 3000× *g* and 4 °C for 10 min to obtain serum. ELISA was used for sIgE and sIgG1 analysis as previously described [20], with slight modifications. Briefly, 100 μL shrimp protein extract (4.8 mg/mL) was used for 96-well plate coating and incubated overnight at 4 °C. The wells were washed with 300 μL TBST buffer (0.1 M Tris, 0.15 M NaCl, 0.05% Tween-20, pH 7.5) and then blocked with 200 μL blocking solution (5% skimmed milk powder in TBST) at 37 °C for 2.5 h. After washing, 30 μL diluted sera (1:10 dilution for IgE, 1:800 dilution for IgG1) were added to each well and incubated at 37 °C for 2 h. After washing, 100 μL of horseradish peroxidase (HRP) conjugated rat anti-mouse IgE secondary antibody (1:5000 dilution, Abcam, UK) or HRP conjugated goat anti-mouse IgG1 heavy chain secondary antibody (1:8000 dilution, Abcam, UK) was added to each well and incubated at 37 °C for 1.5 h. After TBST washing, 100 μL of TMB substrate solution (Beyotime Biotech, Shanghai, China) was added to each well. The plates were incubated at 37 °C for 20 min. The color reactions were stopped with 50 μL stop solution (2 M H_2_SO_4_). The absorbance at 450 nm (OD_450 nm_) was read with Varioskan Flash Spectral Scanning Multimode Reader (Thermo Fisher Scientific, Waltham, USA). The experiment was performed in octuplicate and repeated three times.

### 2.4. Cytokine Measurement

Blood samples were collected as described above at 30 min after stimulation on day 42, and then serum was obtained by the protocol mentioned above. sIgE and sIgG1 were analyzed by ELISA as described above. ELISA Kits (ebioscience, San Diego, USA) were used to determine the serum histamine, IL-4, IFN-γ, and mMCP-1 levels following the manufacturer’s recommendations. The experiment was performed in octuplicate and repeated three times.

### 2.5. Assessment of Clinical Anaphylaxis and Body Temperature

After the shrimp protein extract challenge on day 42, the states of the mice were observed for 30 min, and clinical anaphylaxis was scored. Clinical anaphylaxis was scored as described by Li et al. [21]. In addition, to estimate body temperature, the rectal temperature of mice was measured and recorded 10 min before challenge and 30 min after challenge (at 10 min intervals) to assess their allergic responses. The experiment was performed in octuplicate.

### 2.6. Preparation of Single Cell Suspension of Spleen

On day 42, mice were euthanized by cervical dislocation 1 h after challenge. As previously described, spleens were dissected to prepare single cell suspensions [22]. After filter through 100 μm cell strainer, cell suspension was spun down at 400× *g* 7 min, then, the cell pellet was resuspended in 1 mL ice-cold PBS for subsequent analysis.

### 2.7. Fluorescent Staining for Flow Cytometer Analysis

For phenotypic analysis of DCs, single cell suspension was stained for 30 min on ice in the dark with APC-CD11c, FITC-MHC II, and PerCP/Cyanine5.5-B220; APC-CD11c, FITC-MHC II, and PE/Dazzle 594-CD40; or APC-CD11c, FITC-MHC II, and PE-CD68. Then, FACSAria SORP (BD Biosciences, Australia) was used for fluorescence staining analysis. The experiment was performed in triplicate. The fluorescently conjugated antibodies were used for flow cytometer analysis (see Table 1).

### 2.8. Isolation of Spleen DCs by Fluorescene-Activated Cell Sorting

For DCs sorting, single cell suspension was stained for 30 min on ice in the dark with APC-CD11c and FITC-MHC II (eBioscience, San Diego, USA), then FACSAria SORP was used for sorting CD11c^+^MHC II^+^DCs.

### 2.9. Lipid Extraction and Widely Targeted Lipidomics Analysis

Due to the small number of CD11c^+^MHC II^+^DCs that can be sorted in the spleen of each mouse, we combined the DCs obtained by sorting from three mice as one sample, and detected three samples in each group. Lipid extraction and widely targeted lipidomics analysis was performed by Metware Biotechnology Co., Ltd. (Wuhan, China) according to a previously reported study [23]. Briefly, each sample including 1 × 10^6^ DCs was placed in liquid nitrogen for 2 min, then thawed on ice for 5 min and vortex blended. This step was repeated three times, then the sample was centrifuged at 5000 rpm and 4 °C for 1 min. Each sample was mixed with 1 mL of the extraction solvent (tert-butyl methyl ether: methanol = 3:1, *v*/*v*) containing the internal standard, and 200 μL of deionized water was added after stirring for 15 min. The mixture was vortexed for 1 min and then centrifuged at 12,000 rpm for 10 min at 4 °C. Then, 500 μL of the upper organic layer was collected and evaporated using a vacuum concentrator (Labconco, Kansas City, USA). The dry extract was reconstituted using 200 μL of mobile phase B before LC-MS/MS analysis.

The sample extracts were analyzed using an LC-electrospray ionization (ESI) -MS/MS system (UPLC, ExionLC AD, https://sciex.com.cn/, accessed on 15 May 2021; MS, triple quadrupole-linear ion trap (QTRAP) System, https://sciex.com/, accessed on 15 May 2021). The analytical conditions were as follows: UPLC: column, Thermo Accucore C30 (i.d.2.1 mm × 100 mm, 2.6 μm); solvent system, A: acetonitrile/water (60/40, *v*/*v*, 0.1% formic acid, 10 mM ammonium formate), B: acetonitrile/isopropanol (10/90, *v*/*v*, 0.1% formic acid, 10 mM ammonium formate); gradient program, A/B (80:20, *v*/*v*) at 0 min, 70:30 *v*/*v* at 2 min, 40:60 *v*/*v* at 4 min, 15:85 *v*/*v* at 9 min, 10:90 *v*/*v* at 14 min, 5:95 *v*/*v* at 15.5 min, 5:95 *v*/*v* at 17.3 min, 80:20 *v*/*v* at 17.5 min, 80:20 *v*/*v* at 20 min; flow rate, 0.35 mL/min; temperature, 45 °C; injection volume: 2 μL.

LIT (linear ion trap) and triple quadrupole (QQQ) scans were acquired on a QTRAP-MS, QTRAP LC-MS/MS System, equipped with an ESI Turbo Ion-Spray interface, operating in positive and negative ion modes and controlled by Analyst 1.6.3 software (Sciex, Framingham, USA). The ESI source operation parameters were as follows: ion source, turbo spray; source temperature, 500 °C; ion spray voltage, 5500 V (positive), −4500 V (negative); ion source gas I, gas II, and curtain gas were set at 45, 55, and 35 psi, respectively; and the collision gas was medium. Instrument tuning and mass calibration were performed using 10 and 100 μM polypropylene glycol solutions in the QQQ and LIT modes, respectively. QQQ scans were acquired as multiple reaction monitoring (MRM) experiments with the collision gas (nitrogen) set to 5 psi. Declustering potential and collision energy for individual MRM transitions were determined with further optimization. A specific set of MRM transitions was monitored for each period according to the lipids eluted within this period.

Quality control, qualitative, and quantitative analysis of lipids were according to Yuan et al. [23]. The statistical function prcomp function in R (version 3.6.3) was used to perform unsupervised principal component analysis (PCA). The data were unit variance scaled [24] (i.e., z-score normalization) before unsupervised PCA by setting the prcomp function parameter scale = True. The hierarchical cluster analysis was carried out by R package ComplexHeatmap. Normalized signal intensities of lipids (unit variance scaling) were visualized as a color spectrum. Significantly regulated lipids between groups were determined by variable importance in projection (VIP) ≥ 1 and |log2 fold change| ≥ 1. VIP values were derived from the orthogonal partial least squares discriminant analysis (OPLS-DA) result, which was generated using the R package MetaboAnalystR (version 1.0.1). The data were log transformed (log2) and mean centering was performed before OPLS-DA. In order to avoid overfitting, a permutation test (200 permutations) was performed. The detected lipids were annotated using the Kyoto Encyclopedia of Genes and Genomes (KEGG) compound database (http://www.kegg.jp/kegg/compound/, accessed on 28 May 2021), and the significantly regulated lipids were mapped to the KEGG pathway database [25]. Significantly enriched pathways were determined by the *p*-value of the hypergeometric tests.

### 2.10. RNA Sequence Analysis

Total RNA was extracted from the spleen cells of SA and CK group using TRIzol reagent (Invitrogen, Waltham, USA) according to the manufacturer’s instructions. RNA degradation and contamination monitoring, purity checks, concentration measurements, integrity assessment, and generation of sequencing libraries were performed by Metware Biotechnology Company according to the method described by Han et al. [26]. Index codes were added to attribute sequences of each sample. The library preparations were sequenced on an Illumina HiSeq platform (Illumina, San Diego, USA). The transcriptomic data were available in the GenBank database under the BioProject accession number: PRJNA826371.

Differential expression between the SA and CK groups was analyzed using DESeq2 v1.22.1 [27]. For identifying differentially expressed genes (DEGs), |log2 fold change| ≥ 1 and *p* < 0.05 were used as the screening criteria. The enrichment analysis was performed based on the hypergeometric test. A hypergeometric distribution test was performed with the pathway unit for KEGG. Gsea-3.0.jar was used for gene set enrichment analysis (GSEA). The experiment was performed with three mice in each sample and three samples in each group.

### 2.11. Correlation Analysis of Transcriptome and Lipidome

Correlation analysis was performed on transcriptome and lipidome, and the Pearson correlation coefficient (PCC) between transcriptome and lipidome was calculated using the cor () function in R. Results with |PCC| > 0.8 were selected. To better understand the relationship between genes and lipids related to lipid metabolism and immune inflammation, genes and lipids were simultaneously mapped to KEGG pathway maps.

### 2.12. Dendritic Cell Line DC2.4 Culture, Cell Treatment, and RNA Extraction

Dendritic cell line DC2.4 was purchased from Xiangf bio, Ltd. (Shanghai, China) and cultured in RPMI1640 medium, supplemented with 10% FBS (GIBCO, Waltham, USA), 100 U/mL penicillin (Servicebio, Wuhan, China), and 0.1 mg/L streptomycin (Servicebio, Wuhan, China). To assess the potential effect of triglyceride and ceramide on DC2.4, DC2.4 also was cultured in the above medium with glyceryl trioleate (500 μM, SIGMA, St. Louis, MO, USA) and C16 ceramide (20 μM, Avanti, Birmingham, AL, USA), respectively, for 24 h, and then total RNA was isolated from DC2.4 according to a previously described protocol [28] for real-time reverse transcriptase PCR (RT-qPCR) analysis. The experiment was performed in triplicate and repeated three times.

### 2.13. RT-qPCR

The HiScript III RT SuperMix for qPCR (+gDNA wiper) (Vazyme, Nanjing, China) kit was used for reverse transcription. Furthermore, Taq Pro Universal SYBR qPCR Master Mix Kit (Vazyme, Nanjing, China) was used for RT-qPCR in the Bio-Rad iCycler iQ system (Bio-Rad, USA). RT-qPCR was carried out under the following conditions: 95 °C for 30 s, then 40 cycles (95 °C for 10 s and 60 °C for 30 s). The RT-qPCR primer sequences used for *β-actin*, *Prkcg*, *Pik3r1*, *Akt1*, *Stat3*, *Rela*, *Il4*, *Il12a*, *Cd68*, and *Ptprc* (B220) are shown in Table 2. The relative level (fold change (2^−^^ΔΔCT^) or log2 fold change) for each transcript was calculated. The experiment was performed in triplicate and repeated three times.

### 2.14. Statistics

Data were analyzed using GraphPad Prism built-in tests. Statistical comparisons were performed by unpaired *t*-test or Mann−Whitney test based on the normality of data. Welch’s correction was used for unequal variances. Experimental data are expressed as mean ± SEM. Significance was defined as *p* < 0.05.

## 3. Results

### 3.1. Shrimp Allergic Mouse Model Showed Typical Allergic Reactions

**Figure 1:**
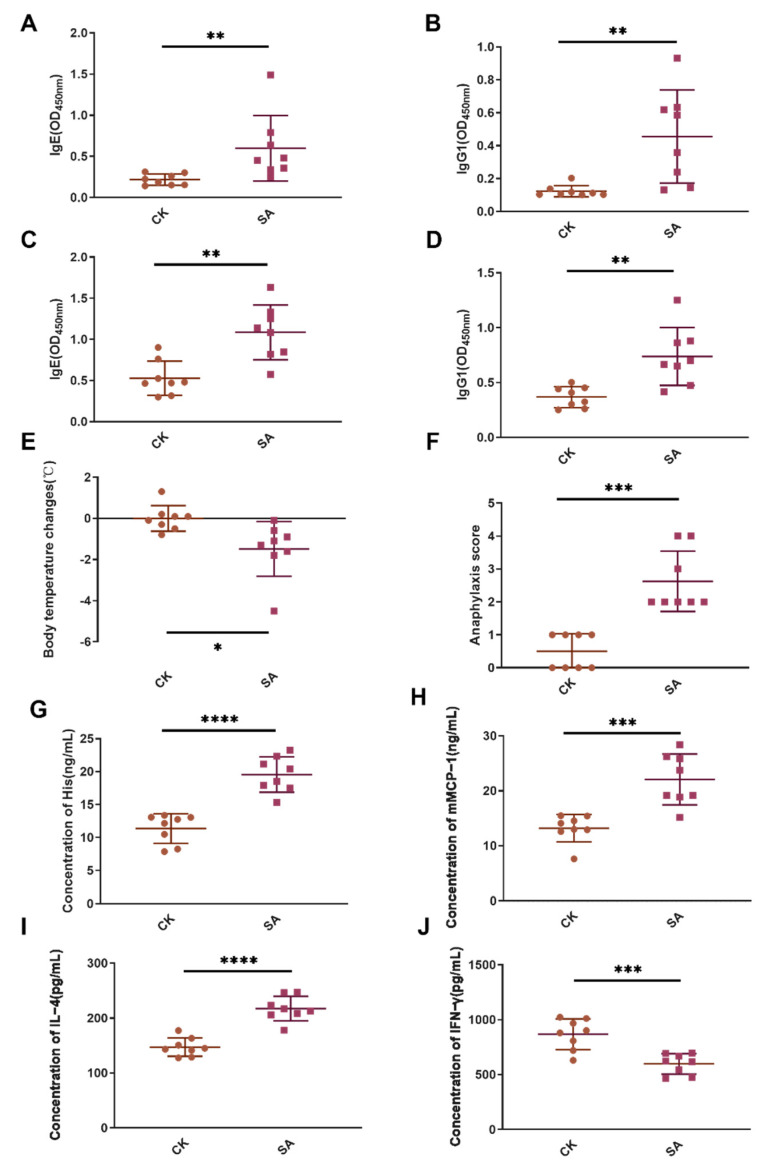
Allergy index analysis of mice in CK and SA groups. (**A**,**B**) sIgE and sIgG1 levels on day 35; (**C**,**D**), sIgE and sIgG1 levels on day 42; (**E**,**F**), body temperature changes and anaphylaxis score after challenge. (**G**–**J**), histamine, mMCP-1, IL-4, and IFN-γ levels in each group. *, *p* < 0.05; **, *p* < 0.01; ***, *p* < 0.001; ****, *p* < 0.0001. CK, control group; SA, shrimp allergy group. Square represent mice from SA group; Circle represent mice from CK group. *n* = 8.

To determine whether the shrimp allergic model was successful, the allergic indexes of mice after modeling were evaluated. On day 35 and 42 sIgE and sIgG1 were detected, and there were significant difference between the CK and SA groups (*p* < 0.01, Figure 1A–D), suggesting that the SA group showed strong antibody response. In this study, we further observed the body temperature and clinical anaphylaxis of mice after challenge. Compared with the CK group, the decrease in body temperature was greater (*p* < 0.05, Figure 1E), and anaphylaxis score was significantly higher (*p* < 0.001, Figure 1F) in the SA group. These results suggested that the SA group showed allergic reactions. The levels of histamine and mMCP-1 in the SA group were significantly higher than in the CK group (*p* < 0.001, Figure 1G,H), indicating severe degranulation in the SA group.

The levels of L-4 and IFN-γ released in SA and CK groups were also detected. In the SA group, levels of IL-4 (Th2 related cytokines) were significantly higher than in the CK group (*p* < 0.0001, Figure 1I), suggesting that shrimp protein can induce the secretion of Th2-type cytokines and promote Th2-type immune response. In addition, the level of IFN-γ in the SA group was significantly lower than in the CK group (*p* < 0.001, Figure 1J), indicating that Th1 immune response in the SA group was suppressed. Taken together, we can conclude that the shrimp allergic model was successfully established, and the phenotype analysis of spleen DCs and fluorescence-activated cell sorting of spleen DCs could be carried out.

### 3.2. SA Affected Lipid Metabolism and Immune Function of Spleen DCs

**Figure 2:**
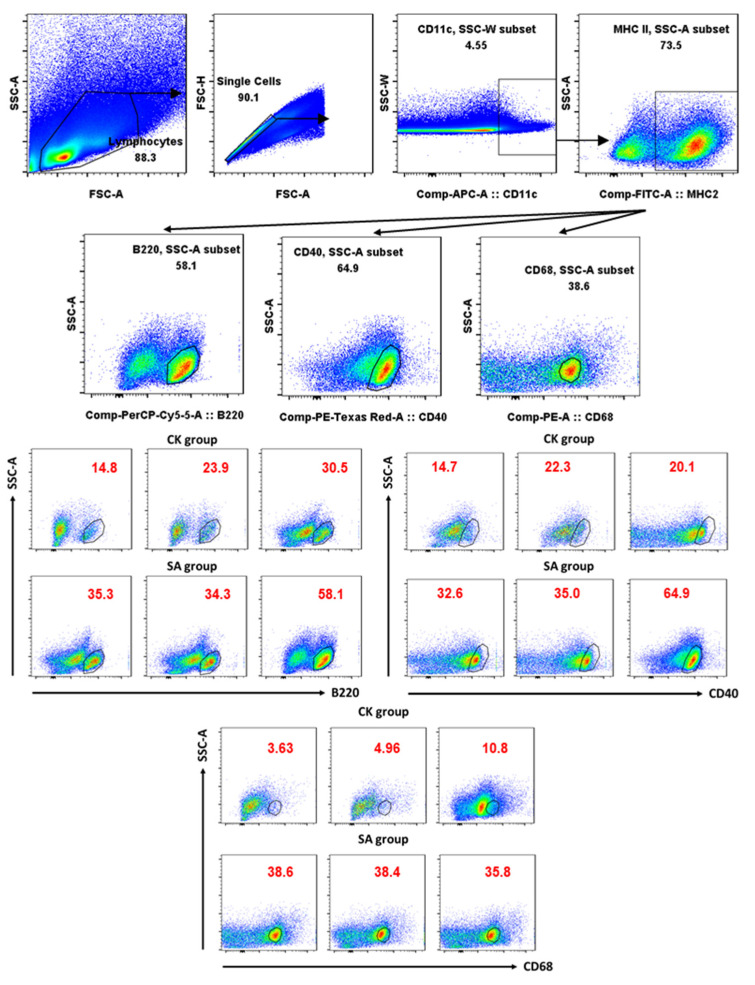
The percentage of B220 (*p* = 0.096), CD40 (*p* = 0.077) and CD68 (*p* < 0.001) expressed DCs. CK, control group; SA, shrimp allergy group. Arrows represent gating logic for flow cytometry analysis. *n* = 3.

To assess whether SA could affect lipid metabolism and immune function of DCs in spleen, the expression of surface receptors related to lipid absorption (CD68) and immune function (B220, CD40) were examined by flow cytometry. As shown in Figure 2, SA spleen DCs exhibited significantly higher expression of lipid scavenger receptors CD68 (*p* < 0.001), and higher expression of plasmacytoid marker B220 and co-stimulatory molecules CD40 with insignificant difference, which indicated the relationship between SA and lipid metabolism and immune function of spleen DCs.

### 3.3. Lipid Profile Distinguished Spleen DCs in SA from Normal

**Figure 3:**
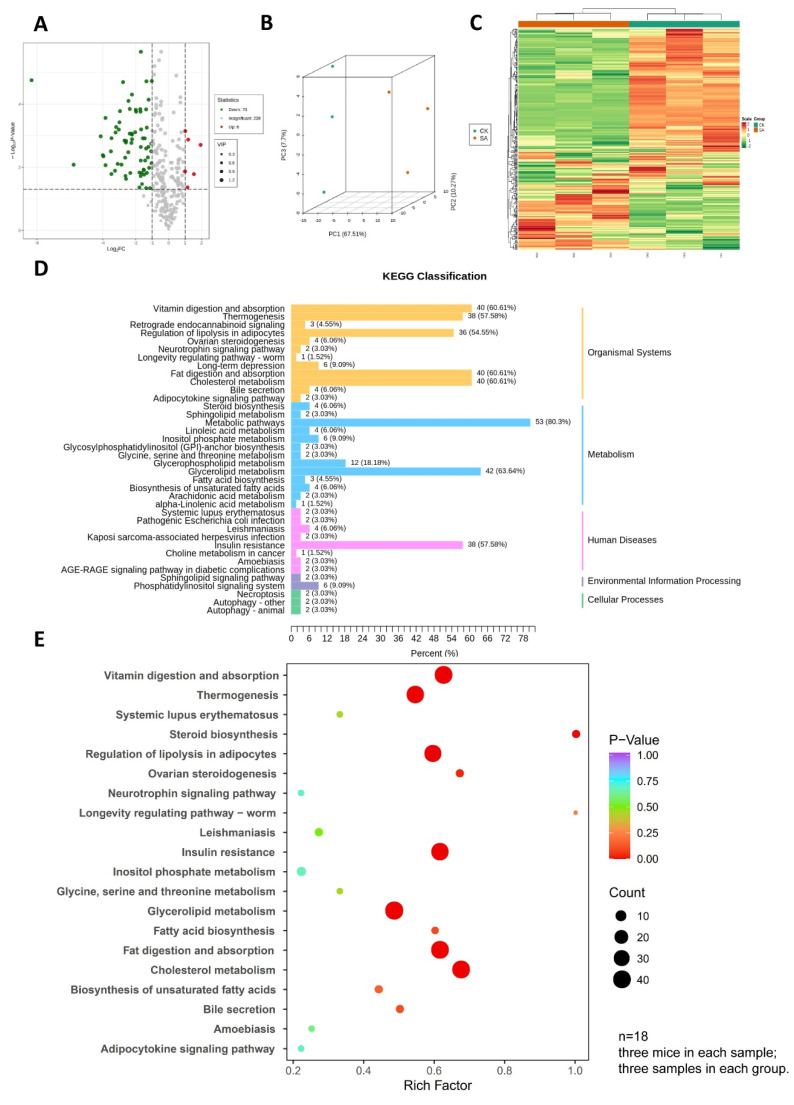
Lipidomic analysis of spleen dendritic cells in control and shrimp allergy groups. (**A**) volcano plot of the 314 lipids profile; (**B**) principal component analysis of the global spleen dendritic cells’ lipidome demonstrated that the two groups (shrimp allergy vs. normal spleen dendritic cells) were clearly distinguished; (**C**) hierarchical clustering heatmap of lipids in normal and shrimp allergy spleen dendritic cells; (**D**,**E**), KEGG pathway enrichment showed the most altered lipid metabolic pathways in shrimp allergy.

To determine the effect of SA on lipid metabolism of spleen DCs, we performed widely targeted lipidomic analysis of DCs sorted from spleen. In total, 314 lipids were identified, and 76 were found to be differentially accumulated in SA spleen DCs compared to normal samples (6 higher and 70 lower) (Figure 3A). Lipidomic data were used for PCA and the results showed that SA and CK groups can be completely distinguished, which indicated that the lipid metabolism in the SA group was clearly different from that in the CK group (Figure 3B). In accordance with PCA, hierarchical clustering analysis and heatmap visualization showed a clear distinction between SA and non-allergic spleen DCs (Figure 3C).

To obtain a global overview of altered biochemical processes of differentially accumulated lipids (DALs), we performed KEGG pathway enrichment analysis and the results were classified according to the pathway types. These functional approaches showed that alterations in SA spleen DCs’ lipidome had the highest impact on vitamin digestion and absorption, fat digestion and absorption, cholesterol metabolism, glycerolipid metabolism, and insulin resistance (*p* < 0.05, Figure 3D,E).

### 3.4. Functional Enrichment of DEGs in SA

**Figure 4:**
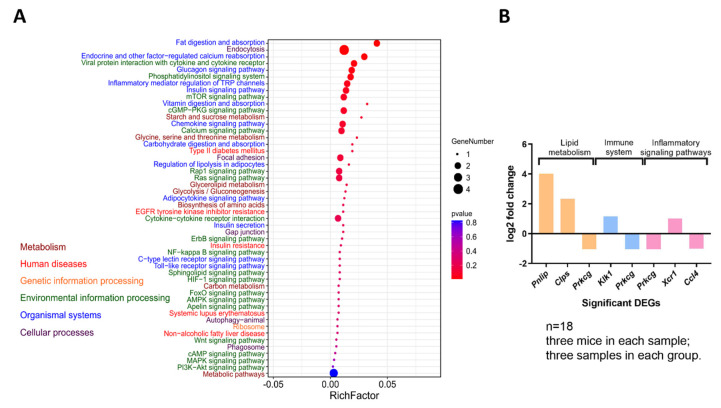
Functional enrichment of DEGs in SA. (**A**) Biological processes and associated pathways that are important for shrimp allergic pathogenesis and development are plotted in bubble chart. The *y*-axis is KEGG pathway, *x*-axis is rich factor. (**B**) DEGs associated with lipid metabolism, immune system, and inflammatory signaling pathways in SA group.

Compared to the CK group, the SA group showed 252 DEGs. Out of the 252 DEGs, 200 and 52 were up- and downregulated, respectively. To understand the biological implication of DEGs, pathways significantly affected in the SA group were identified by functional enrichment analysis. In this study, pathways were shortlisted based on their relevance to the biological processes that are important for shrimp allergic pathogenesis and development and the results showed that SA significantly affected lipid metabolism, immune system, and inflammatory signaling pathways: for example, fat digestion and absorption and phosphatidylinositol signaling system in lipid metabolism; endocytosis, endocrine and other factor-regulated calcium reabsorption, and glucagon signaling pathway in immune system. Viral protein interaction with cytokine and cytokine receptors and inflammatory mediator regulation of TRP channels in inflammatory signaling pathways were significantly affected in the SA group (*p* < 0.05, Figure 4A).

Protein kinase C gamma type (PRKCG), an important enzyme that can be activated by calcium and second messenger diacylglycerols (DAGs), was found involved in all of the three physiological system (lipid metabolism, immune system, and inflammatory signaling pathways) (Figure 4B). These results indicated that lipid metabolism was involved in SA and might have an important impact on the inflammatory response and immune system.

### 3.5. GSEA of B220, CD40, and CD68 Related Gene Sets

**Figure 5:**
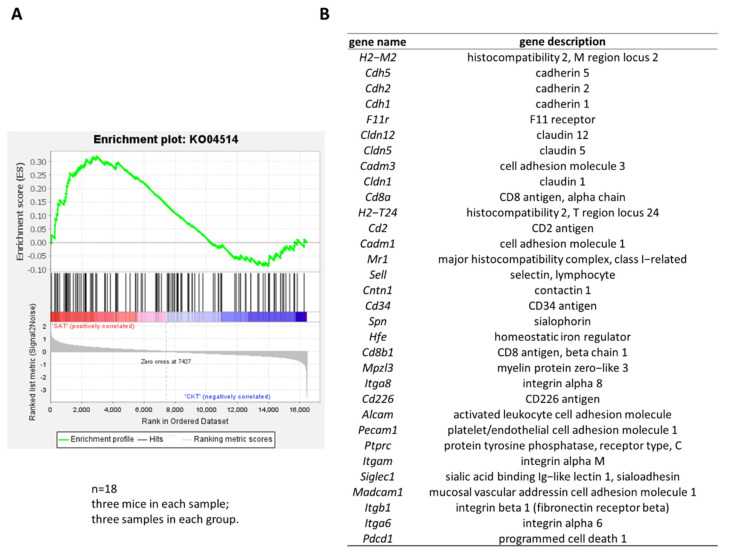
GSEA of B220, CD40, and CD68 related gene sets. (**A**) Gene set enrichment analysis of ko04514; (**B**) genes contribute to the leading-edge subset within ko04514 gene set.

To evaluate the potential molecular mechanism of B220, CD40, and CD68 high expression in SA spleen DCs, gene sets related to B220, CD40, and CD68 in the KEGG signaling pathway (B220 (ko04514, ko05340), CD40 (ko04514, ko05340), CD68 (ko04142)) (Table S1) were selected for GSEA [29]. The results showed that the gene set of ko04514 was upregulated in SA (Figure 5A, normalized enrichment score = 1.43, nominal *p* value = 0.028, FDR = 0.052), and gene sets ko05340 and ko04142 were not significantly enriched, which indicated that SA affected the expression profile of B220 and CD40. Thirty-two genes contributed to the leading-edge subset within the ko04514 gene set, depicting histocompatibility, cadherin, cell adhesion molecule, histocompatibility 2-T region locus 24, activated leukocyte cell adhesion molecule, and protein tyrosine phosphatase receptor type C (Figure 5B).

### 3.6. Correlation Analysis Based on Transcriptome and Lipidome

**Figure 6:**
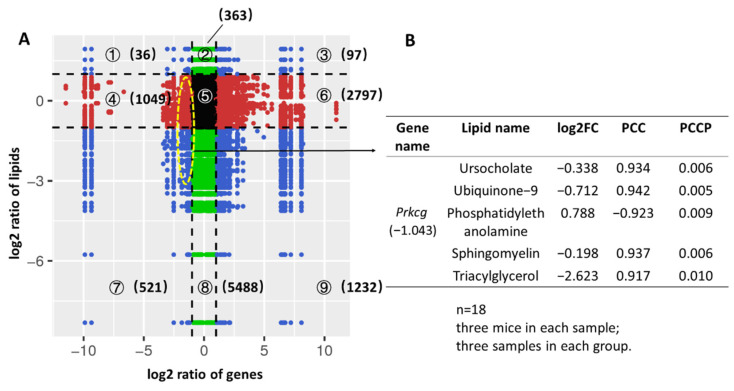
Latent associations among genes and lipids. (**A**) Quadrant diagram represents the association of genes and lipids. The *x*-axis and *y*-axis represent the log2 ratio of genes and the log2 ratio of lipids, respectively; black dotted lines represent the threshold of significant difference; black dots represent that genes and lipids were not significantly changed; green dots represent that lipids were significantly changed and genes were not significantly changed; red dots represent that genes were significantly changed and lipids were not significantly changed; blue dots represent that genes and lipids were significantly changed. The number in brackets represents the number of dots; (**B**) lipids associated with *Prkcg* (|PCC| > 0.8).

In Section 3.4, we found that *Prkcg* was involved in all of the three physiological systems. To investigate the latent associations among *Prkcg* and lipids, correlation analysis were carried out based on transcriptome and lipidome. The correlation analysis results with |PCC| > 0.8 and the correlation coefficient of *Prkcg* with lipids (|PCC| > 0.8) are shown in Figure 6. The ① and ⑨ quadrants indicate that DEGs and DALs have inconsistent regulation trends, the DALs may be negatively regulated by DEGs; the ② and ⑧ quadrants indicate that the genes have not changed, and lipids are up- or downregulated; the ③ and ⑦ quadrants indicate that DEGs and DALs have consistent regulatory trends, the DALs may be positively regulated by DEGs; the ④ and ⑥ quadrants indicate that the lipids have not changed, and genes are up- or downregulated; the ⑤ quadrant indicates that the genes and lipids are not significantly changed. Through correlation analysis, we found that the correlation of *Prkcg* and triacylglycerol (TAGs) was in ⑦ quadrant and had the maximum |log2 fold change| (2.623), suggesting that *Prkcg* was positively correlated with TAGs. However, the other lipids (|PCC| with *Prkcg* was greater than 0.8) were not significantly accumulated in SA.

### 3.7. Pathway Analysis Based on Lipidome and Transcriptome

To anchor the lipids and genes related to lipid metabolism and immune inflammation in SA spleen DCs, genes and lipids were simultaneously mapped to KEGG pathway maps (Tables S2 and S3). The anchored DEG was validated by RT-qPCR (Figure S1).

**Figure 7:**
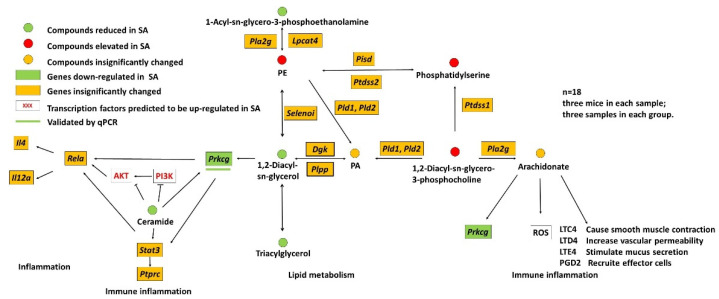
Transcriptome and lipidome integrated pathway diagram presents alterations of lipids and genes related to lipid metabolism and immune inflammation in SA spleen DCs. PE, phosphatidylethanolamine; PA, phosphatidate acid.

As shown in Figure 7, of a total of nine specific lipids, three increased (phosphatidylethanolamine, phosphatidylserine, and 1,2-diacyl-sn-glycero-3-phosphocholine) and four decreased (1-acyl-sn-glycero-3-phosphoethanolamine, TAGs, DAGs, and ceramide) and were mapped in the pathway diagram. One of the most eye-catching features in this diagram is the activated metabolic conversion from TAGs to DAGs and the transmission of lipid metabolism to immune inflammation (from TAGs and ceramide to *Prkcg*) in SA spleen DCs. A comprehensive analysis of the annotated pathway maps ko00561 (Figure S2), ko04070 (Figure S3), ko04931 (Figure S4), ko04722 (Figure S5), ko04071 (Figure S6), and ko04920 (Figure S7) showed that DAGs and TAGs were positively correlated with *Prkcg* (Figures S2 and S3). Ceramide inhibited AKT and PI3K (Figures S4 and S5). In addition, ceramide was positively correlated with *Prkcg* (Figure S6) and *Prkcg* was positively correlated with NF-κB (Figure S7). In this study, the transcriptome and lipidome showed that *Prkcg*, TAGs, and ceramide were decreased in SA, which was consistent with the positive relationship between *Prkcg* with TAGs and ceramide. We also found that the transcription factors (AKT, PI3K) were upregulated in SA, which was also consistent with the negative relationship between ceramide with AKT and PI3K. Highly expressed AKT and PI3K could stimulate the NF-κB signaling pathway (Figure S5), and then cause inflammatory responses. Comprehensively, changes in lipid metabolism (including decreases in TAGs, ceramide, and DAGs) could cause immune inflammation.

### 3.8. Glyceryl Trioleate and C16 Ceramide Affected the Immune Function of Dendritic Cell Line DC2.4

**Figure 8:**
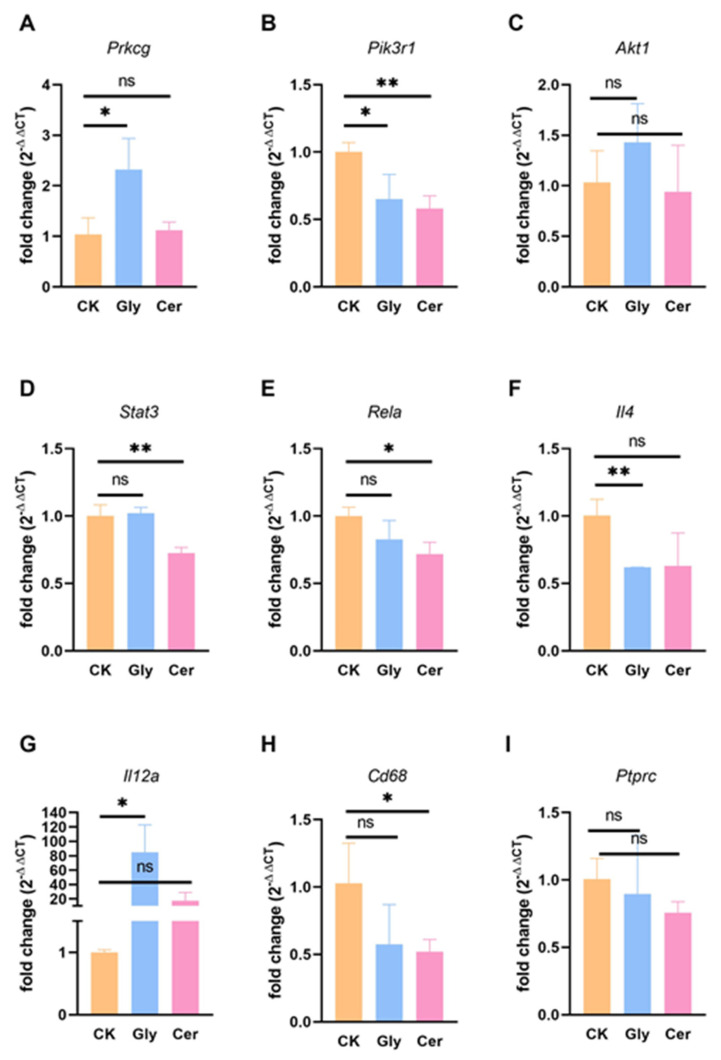
Effects of glyceryl trioleate and C16 ceramide treatment on the expression of *Prkcg* (**A**), *Pik3r1* (**B**), *Akt1* (**C**), *Stat3* (**D**), *Rela* (**E**), *Il4* (**F**), *Il12a* (**G**), *Cd68* (**H**), and *Ptprc* (**I**) in DC2.4. DC2.4 was treated with glyceryl trioleate (500 μM) or C16 ceramide (20 μM) for 24 h, and then the expression of *Prkcg*, *Pik3r1*, *Akt1*, *Stat3*, *Rela*, *Il4*, *Il12a*, *Cd68*, and *Ptprc* were detected by RT-qPCR. *, *p* < 0.05; **, *p* < 0.01; ns, no significance. CK, control group; Gly, glyceryl trioleate treated group; Cer, C16 ceramide treated group. *n* = 3.

To further determine the effect of TAG and ceramide on the immune function of DCs, we treated the dendritic cell line DC2.4 with glyceryl trioleate and C16 ceramide, and then the expression of *Prkcg*, *Pik3r1*, *Akt1*, *Stat3*, *Rela*, *Il4*, *Il12a*, *Cd68*, and *Ptprc* was detected by RT-qPCR. Glyceryl trioleate treatment significantly upregulated *Prkcg* expression, whereas C16 ceramide upregulated *Prkcg* expression with insignificant difference (Figure 8A). Both glyceryl trioleate and C16 ceramide treatments caused a significant decrease in the expression of *Pik3r1*, but not *Akt1* (Figure 8B,C). In addition, C16 ceramide significantly reduced the expression of *Stat3* and *Rela* in DC2.4, while glyceryl trioleate did not (Figure 8D,E). Moreover, glyceryl trioleate significantly decreased the expression of *Il4*, a cytokine that induces Th2 cell differentiation, and significantly increased the expression of *Il12a*, a cytokine that induces Th1 differentiation. Although ceramide also slightly decreased DC2.4 *Il4* expression and increased *Il12a* expression, there were no significant differences (Figure 8F,G). This suggested that glyceryl trioleate can improve the Th2 immune skew that causes SA. Furthermore, C16 ceramide significantly decreased the expression of *Cd68*, a lipid scavenger receptor of DCs (Figure 8H). However, glyceryl trioleate and C16 ceramide slightly decreased the expression of *Ptprc* (B220) in DC2.4 with no significant difference (Figure 8I). These results suggested that glyceryl trioleate and C16 ceramide can affect the immune function of DCs.

## 4. Discussion

Shrimp is a delicacy widely eaten by consumers worldwide. However, SA is often associated with severe allergic reactions [30], including life-threatening anaphylaxis [31]. Therefore, SA has become a major public health concern. Presently, lipid metabolism of DCs, the main immune cells involved in allergic diseases, has been found to be altered in asthma and tumors [14,17,32]. Systematic elucidation of lipid metabolism and its correlation with immune function in SA spleen DCs is of critical significance.

In this study, the SA group showed allergic responses, such as increased sIgE, sIgG1, histamine, mMCP-1, anaphylaxis score, and IL-4, decreased IFN-γ and body temperature, which were consistent with previous study [33,34]. DCs as antigen-presenting cells participate in allergies by expressing B220 and CD40 [35,36]. According to the research of Ibrahim et al. [9], high-lipid-content DCs exhibited modestly higher expression of the lipid scavenger receptor CD68. Thus, the levels of functional markers (B220, CD40, and CD68) of DCs in spleen were also determined by flow cytometry in this study. Populations of CD11c^+^MHC II^+^CD68^+^DCs increased in SA mice, suggesting an important role of lipid metabolism (high CD68 expression) in SA spleen DCs.

Widely targeted lipidomics revealed a massive change in lipid levels with accumulations of many glycerophospholipids and depletion of TAGs, DAGs, and ceramide. These changes, in association with a decreased expression of genes involved in lipid metabolism (*Prkcg*), were suggestive of an altered lipid metabolism in SA spleen DCs. Glycerophospholipids have been experimentally shown to be involved in allergic sensitization, such as phospatidylcholine [37], which was also increased in this study. Currently, there are few studies on the role of TAGs in spleen DCs in food allergies. A previous report indicated that maternal plasma TAGs are important for food allergy outcomes in the offspring [38]. Maternal TAGs with 41–52 carbons and 0–4 double bonds are associated with greater risk of food allergies, whereas TAGs with 56–60 carbons and 5–12 double bonds are significantly associated with lower risk of food allergies in offspring [39]. In this study, the carbon chains of differentially accumulated TAGs were 40–54 and double bonds were 0–7. However, TAGs were downregulated in the SA group, which was contrary to the finding of Hong et al. [39]. Therefore, the role of TAGs in SA spleen DCs needs further research. DAGs, involved in numerous cell signaling cascades that support glycerolipid biosynthesis and regulate protein kinase C (PKC) [40], were downregulated in the SA group, which was consistent with the finding of Bao et al. [41]. PKCγ (encoded by *Prkcg* gene), a serine- and threonine- specific protein kinase that can be activated by DAGs, is the main enzyme related to the metabolism and immune function of DAGs. Winkler et al. [42] reported that increased PKCγ activity impairs dendritic development. In this study, the levels of *Prkcg* and DAGs were downregulated, which may affect the dendritic development of DCs, and then affect the immune function of DCs in SA.

Through KEGG enrichment analysis of DEGs, we did not find DEGs related to B220, CD40, and CD68. Therefore, we performed GSEA analysis by all genes on ko04514, ko05340, and ko04142, which related to B220, CD40, and CD68. We found that the gene set of ko04514 (B220, CD40) was upregulated in SA, and the gene set of ko04142 (CD68) was not significantly enriched. A possible explanation for this might be that translation occupies a major position in the expression of CD68, which was consistent with the finding of Wang et al. [43].

To investigate the effects of lipid metabolism on immune function of SA spleen DCs, pathway analysis based on lipidome and transcriptome was performed. Briefly, lipid metabolism influenced immune and inflammatory signaling pathways through the regulation of *Prkcg* by DAGs, TAGs, and ceramide. The binding of DAGs to a conserved C1 domain in PKC leads to the plasma membrane translocation and activation of the latter [44]. Studies have reported that PKC mediates NF-κB activation [45,46]. In our study, *Prkcg* was downregulated and *Rela* was insignificantly changed. It was inferred that PKC may function through the dissociation of NF-κB in SA spleen DCs as a pathway map (Figure S7) presented, but had no effect on, the expression of *Rela*. Kitatani et al. [47] and Kim et al. [48] have reported that ceramide mediates the PI3K/AKT pathway inactivation. In our study, ceramide was downregulated and PI3K/AKT was upregulated. It was inferred that ceramide mediates the PI3K/AKT inactivation in SA spleen DCs, which was consistent with previous study [47,48]. PI3K/AKT has been suggested to function as an IKK (IκB kinase) kinase [49,50]. NF-κB regulates diverse cellular activities associated with inflammation, and innate and adaptive immune responses, which play an important role in allergic disease [51,52,53,54]. Studies also have shown that ceramide has certain interactions with PKC [55] and STAT3 [56]. PKC and ceramide can also activate the STAT3 pathway and then act on NF-κB and PTPRC, thus regulating allergic reaction [57,58,59]. Therefore, DAGs, TAGs, and ceramide may affect SA through *Prkcg*, which may have great significance in regulating inflammation related to SA.

We validated the lipidome and transcriptome based pathway analysis by cellular experiments in vitro. The expression of *Prkcg* in DC2.4 was significantly increased after treatment with exogenous glyceryl trioleate, which was consistent with the positive correlation between TAGs and *Prkcg* as determined by the lipidomics and transcriptomics in this study. Similarly, exogenous glyceryl trioleate and C16 ceramide treatment caused a significant decrease in *Pik3r1*, which was also consistent with the lipidome and transcriptome. Interestingly, C16 ceramide significantly reduced the expression of *Stat3* and *Rela*, whereas glyceryl trioleate did not. However, glyceryl trioleate caused significant changes in the expression of *Il4* and *Il12a*. A possible explanation for this might be that protein expression, activation or nuclear translocation of RELA plays a crucial role in the expression of *Il4* and *Il12a* [60,61,62], and the specific relationship between *Prkcg* and *Rela* in SA spleen DC needs further research. C16 ceramide treatment significantly reduced the expression of *Cd68* in DC2.4, and both glyceryl trioleate and C16 ceramide treatment slightly reduced the expression of *Ptprc* (B220), all of which were consistent with the negative correlation between lipids (TAGs and ceramides) and phenotypes (CD68 and B220) in lipidomics and cell phenotype results in this study. These data provide evidence for further investigation of the exact mechanism by which TAGs and ceramide affect DC immune function in SA.

## 5. Conclusions

In conclusion, shrimp protein extracts could induce remarkable allergic reactions and noteworthy alteration of lipids in spleen DCs, demonstrating that shrimp allergen sensitization might cause disturbance of lipid metabolism, which further confirmed the relationship between SA and lipid metabolism. The pathway analysis based on transcriptome and lipidome indicated the transmission of lipid metabolism to immune inflammation (from TAGs and ceramide to *Prkcg*) in SA spleen DCs, and the molecular mechanism between lipid metabolism and immune function in DCs was preliminarily studied via in vitro cellular experiments. These results indicated that exogenous glyceryl trioleate and ceramide may have a regulatory effect on the immune function of DCs, and improve the Th2 immune skew induced by shrimp allergenic protein, thereby mitigating SA. This has implications for mitigating SA through dietary supplementation with specific glyceryl trioleate and ceramide. However, further studies are needed to reveal the exact mechanism and provide convincing evidence for the application of lipid metabolism regulation as anti-allergy methods based on DCs.

## Figures and Tables

**Table 1:** Fluorescently conjugated antibodies for flow cytometry.

Surface Antigen	Clone	Source
CD11c	N418	eBioscience
MHC class II	M5/114.15.2	eBioscience
B220	RA3-6B2	eBioscience
CD40	3/23	Biolegend
CD68	FA-11	Biolegend

**Table 2:** Primer sequences used for RT-qPCR.

Primer	Primer Sequences	
	Forward	Reverse
*β-actin*	AAGTGTGACGTTGACATCCGTAAAG	CAGCTCAGTAACAGTCCGCCTAGA
*Prkcg*	CTCCGACGAACTCTATGCCATCAAG	CCAATGCCAGGACACGCTTCTC
*Pik3r1*	TGTGGCACAGACTTGGTGTT	TTCTTCCCTTGAGATGTCTCCC
*Akt1*	CCGCCTGATCAAGTTCTCCT	TTCAGATGATCCATGCGGGG
*Stat3*	TGTCAGATCACATGGGCTAAAT	GGTCGATGATATTGTCTAGCCA
*Rela*	AGACCCAGGAGTGTTCACAGACC	GTCACCAGGCGAGTTATAGCTTCAG
*Il4*	TACCAGGAGCCATATCCACGGATG	TGTGGTGTTCTTCGTTGCTGTGAG
*Il12a*	GACCTGTTTACCACTGGAACTA	GATCTGCTGATGGTTGTGATTC
*Cd68*	GAAATGTCACAGTTCACACCAG	GGATCTTGGACTAGTAGCAGTG
*Ptprc*	GTTATCCACGCTGCTGCCTCAC	TTGGCTGCTGAATGTCTGAGTGTC

## Data Availability

All the data regarding this work are presented in this manuscript.

## Supplementary Materials

The following supporting information can be downloaded at https://www.mdpi.com/article/10.3390/foods11131882/s1. Figure S1: RT-qPCR validation of anchored DEG in pathway analysis. The data are presented as mean ± SEM; Figure S2: Annotated pathway map ko00561; Figure S3: Annotated pathway map ko04070; Figure S4: Annotated pathway map ko04931; Figure S5: Annotated pathway map ko04722; Figure S6: Annotated pathway map ko04071; Figure S7: Annotated pathway map ko04920; Table S1: Gene sets of ko04514, ko05340, and ko04142; Table S2: Lipidomic data set; Table S3: Transcriptomic dataset.
