# Supplementary figures and images for: Widely Targeted Lipidomics and Transcriptomics Analysis Revealed Changes of Lipid Metabolism in Spleen Dendritic Cells in Shrimp Allergy
Source: Foods. 2022 Jun 25;11(13):1882. doi: 10.3390/foods11131882 (PMC9265612; doi:10.3390/foods11131882)

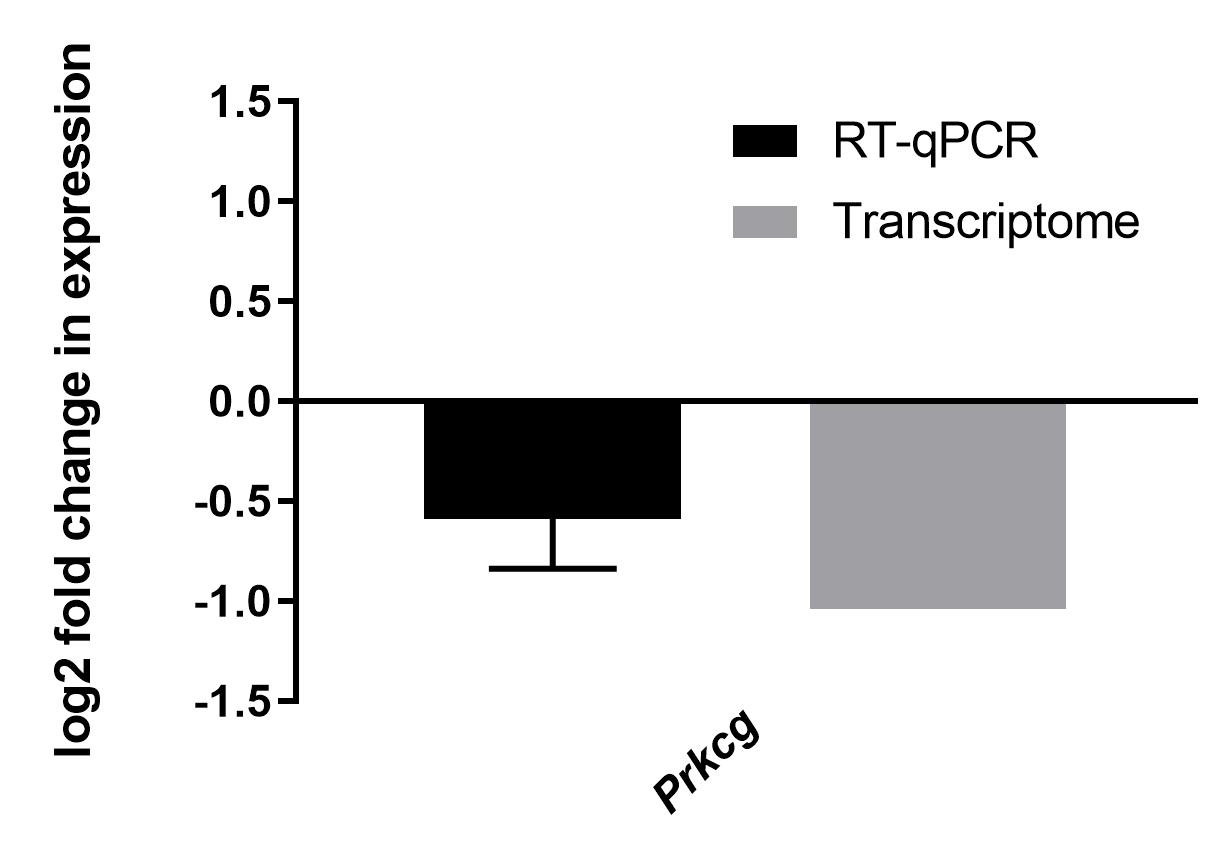

Supplement: Supplementary file 1 [file foods-11-01882-s001.zip › Figure S1.png]

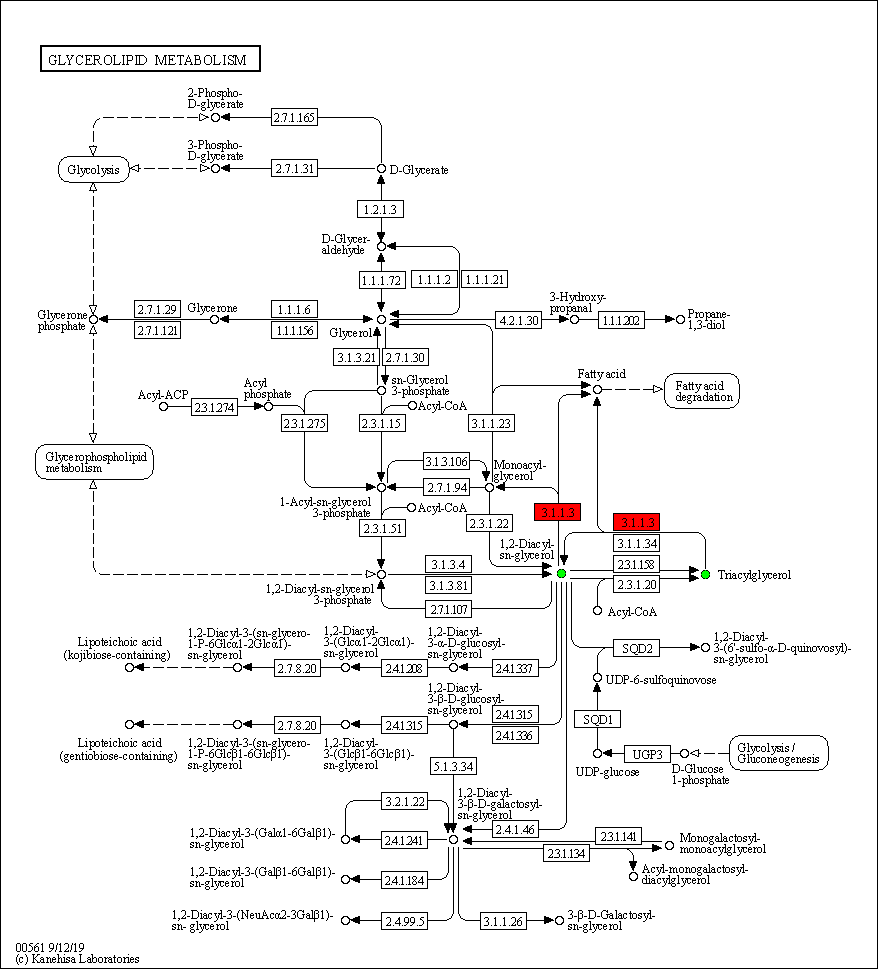

Supplement: Supplementary file 1 [file foods-11-01882-s001.zip › Figure S2.png]

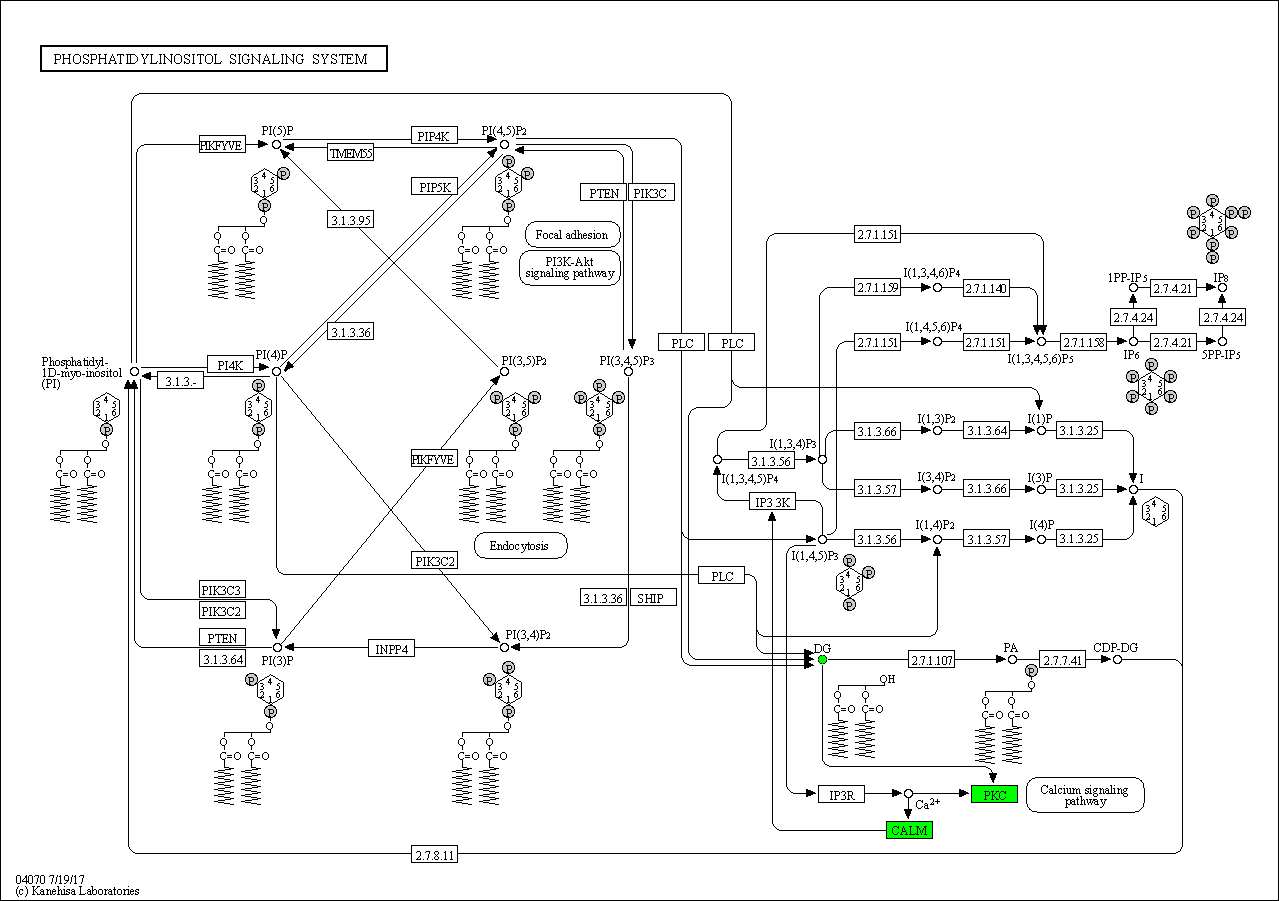

Supplement: Supplementary file 1 [file foods-11-01882-s001.zip › Figure S3.png]

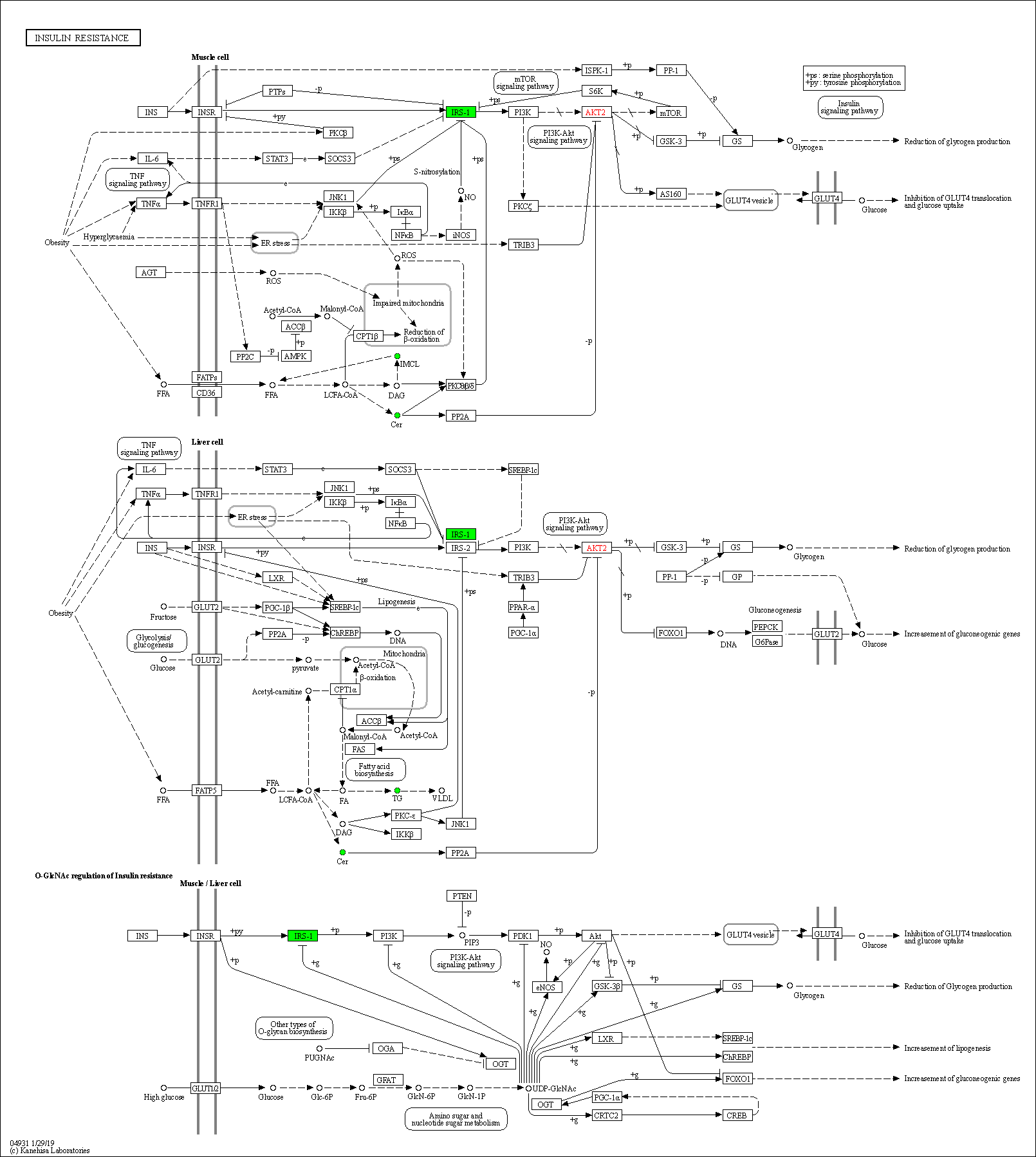

Supplement: Supplementary file 1 [file foods-11-01882-s001.zip › Figure S4.png]

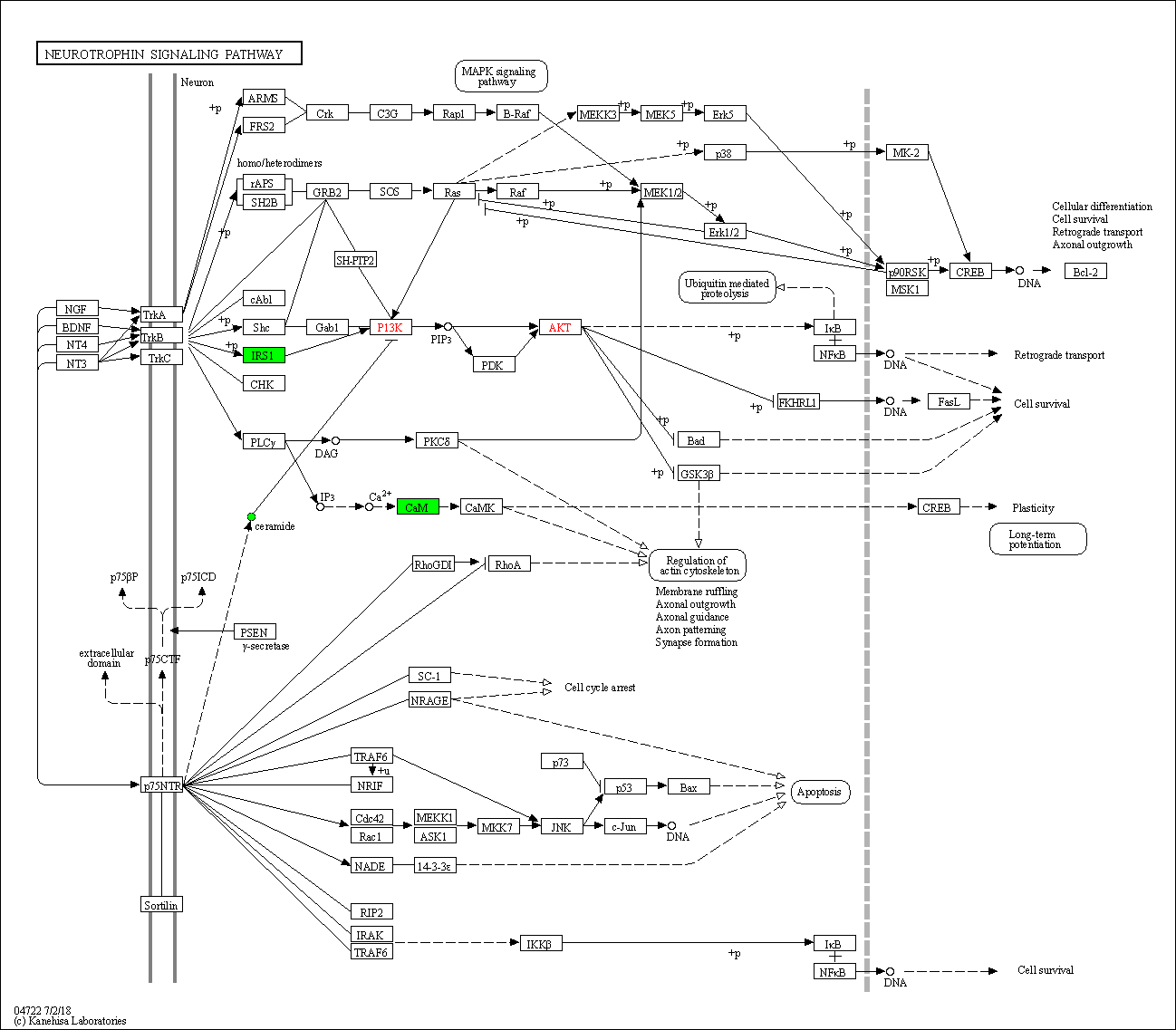

Supplement: Supplementary file 1 [file foods-11-01882-s001.zip › Figure S5.png]

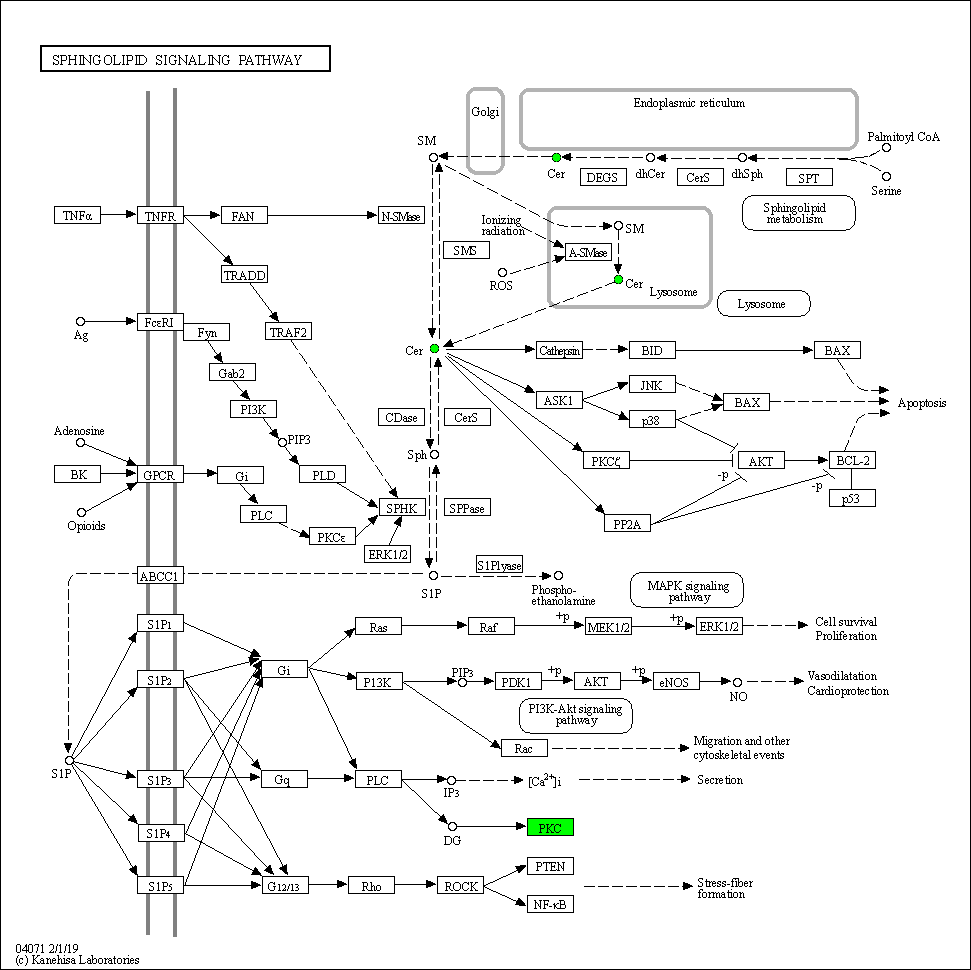

Supplement: Supplementary file 1 [file foods-11-01882-s001.zip › Figure S6.png]

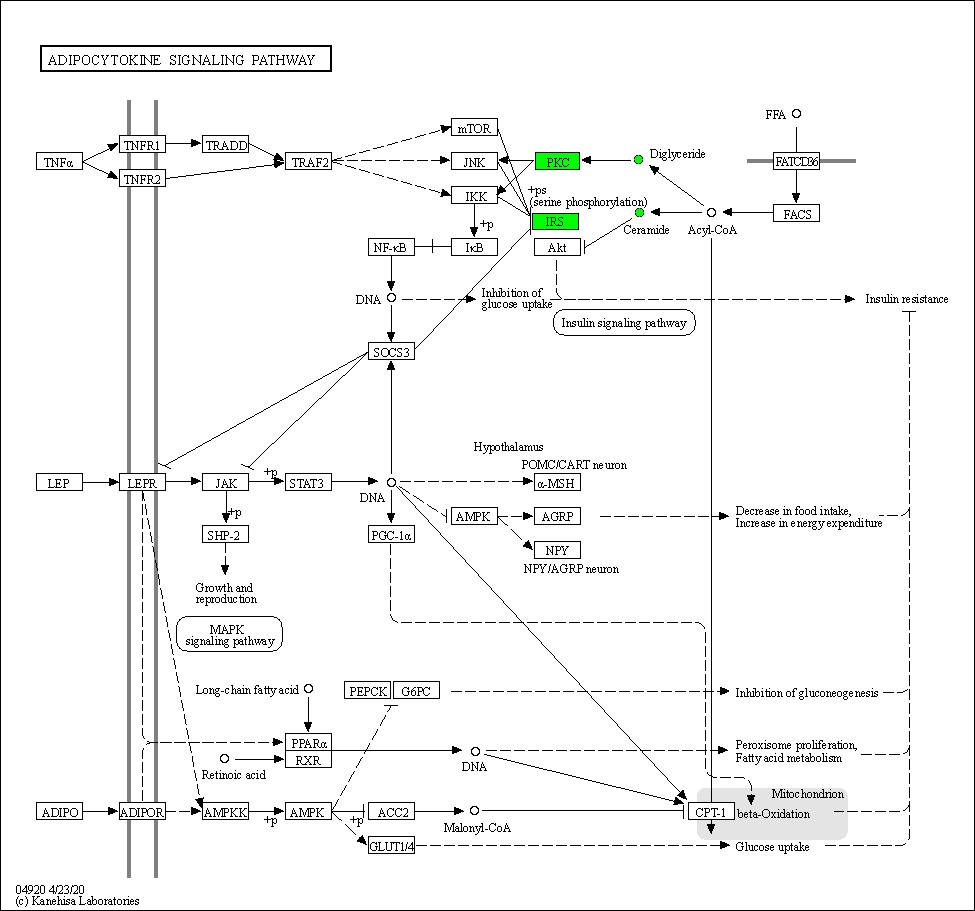

Supplement: Supplementary file 1 [file foods-11-01882-s001.zip › Figure S7.png]
